# US Adults’ Perceptions, Beliefs, and Behaviors towards Plant-Rich Dietary Patterns and Practices: International Food Information Council Food and Health Survey Insights, 2012–2022

**DOI:** 10.3390/nu15234990

**Published:** 2023-12-01

**Authors:** Katherine Consavage Stanley, Valisa E. Hedrick, Elena Serrano, Adrienne Holz, Vivica I. Kraak

**Affiliations:** 1 Department of Human Nutrition, Foods, and Exercise, Virginia Polytechnic Institute and State University (Virginia Tech), Blacksburg, VA 24061, USA; vhedrick@vt.edu (V.E.H.); serrano@vt.edu (E.S.); vivica51@vt.edu (V.I.K.); 2 Virginia Family Nutrition Program, Virginia Tech, Blacksburg, VA 24061, USA; 3 School of Communication, Virginia Tech, Blacksburg, VA 24061, USA; holz@vt.edu

**Keywords:** sustainable diets, sustainability, eating behavior, red meat, plant-based meat alternatives, planetary health

## Abstract

Expert groups recommend that populations adopt dietary patterns higher in whole, plant-based foods and lower in red and processed meat as a high-impact climate action. Yet, there is limited understanding of populations’ willingness to adopt plant-rich dietary patterns. This study examined United States (US) adults’ perceptions, beliefs, and behaviors towards plant-rich dietary patterns and practices over a decade. Fifteen questions from the International Food Information Council’s Food and Health Surveys (2012–2022) were analyzed across four sustainability domains (i.e., human health, environmental, social, and economic domains). Most respondents had favorable perceptions of environmentally sustainable food and beverages, but sustainability influenced less than half of consumers’ purchase decisions. Plant-rich dietary pattern adherence increased across survey years (12.1% [2019] to 25.8% [2022], *p* < 0.001). One-quarter (28.1%) of Americans reported reducing their red meat intake over 12 months (2020–2022). Yet, another 15.5% reported greater red meat intake, and 18.8% reported greater plant-based meat alternative (PBMA) intake over 12 months. The percentage of respondents who reported greater red meat and PBMA consumption in the previous 12 months significantly increased across the years surveyed (2020–2022, *p* < 0.05). IFIC Survey findings highlight growing US consumer awareness of health, environmental, and social sustainability but low adoption of plant-rich dietary patterns and practices. Government leadership and coordinated actions by health professionals, civil society, and businesses are needed to educate and incentivize Americans to adopt plant-rich dietary behaviors, and greater industry transparency is needed to show how food and beverage products support human and planetary health.

## 1. Introduction

The United States’ (US) food system and the average American dietary pattern are not sustainable for supporting long-term human and planetary health and societal well-being [1,2]. There is growing consensus that sustainable diets support nutrition security and human health, environmental and ecological health, social equity, and economic prosperity [3,4,5]. The Food and Agriculture Organization of the United Nations (FAO) and the World Health Organization (WHO) emphasize that sustainable diets “promote all dimensions of individuals’ health and wellbeing; have low environmental pressure and impact; are accessible, affordable, safe, and equitable; and are culturally acceptable” to support current and future generations [6].

Reducing human consumption of red and processed meats (RPM) and shifting people toward dietary patterns higher in minimally processed, whole-plant-based foods (i.e., pulses, legumes, whole grains, nuts, seeds, fruits, and vegetables) is a high-impact action that can mitigate the food system’s impact on climate change [1,7,8,9]. This strategy has been recommended by US and international expert bodies to promote human and planetary health [6,10,11,12,13].

**Figure 1:**
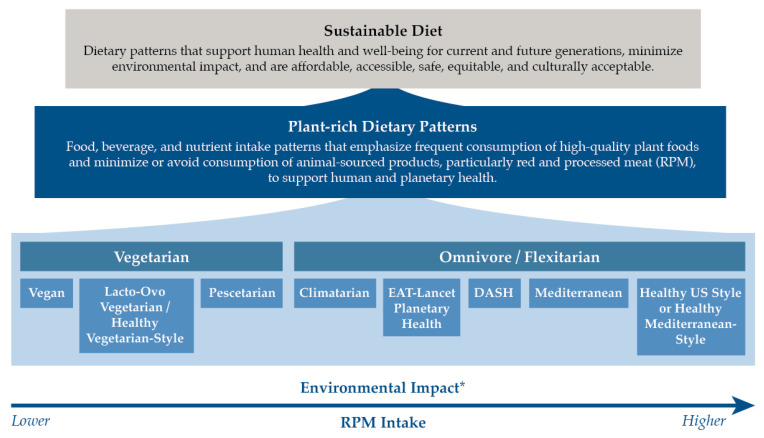
Plant-rich dietary patterns that support sustainable dietary transitions for US adults [6,7,8,10,12,20]. * The environmental impact estimate is based on the level of animal-sourced protein intake, particularly RPM intake, as there is substantial evidence to suggest that dietary patterns high in plant-based foods and low in or free from animal-sourced foods have lower greenhouse gas emissions and land use. These patterns may also have lower water and energy use, although this depends on the types of plant-based foods consumed.

The current Western diet followed by many Americans is characterized by excessive intake of sugary beverages, meat, refined grains, and highly processed foods rich in added sugars and sodium, as well as low intake of fruits, vegetables, nuts and seeds, whole grains, and seafood [8,14,15]. The high US consumer demand for and overconsumption of red meat (i.e., beef, pork, and lamb) and processed meats is of particular concern, as diets rich in these products are linked to an increased risk of type 2 diabetes, heart disease, stroke, and colorectal cancer in individuals and populations [16,17,18]. The large-scale industrialized agricultural production of beef in the US contributes to environmental degradation, as it requires significant water and land use compared to plant-based foods, and produces substantial greenhouse gas emissions, especially methane, that negatively impact the climate [8,19,20]. Many US dietary patterns align with the broader definition of a sustainable diet devised by the FAO and WHO (Figure 1). These patterns can collectively be described as plant-rich dietary patterns and are associated with lower NCD risks and lower environmental impacts (or equivalent, in the case of the Healthy US-Style) compared to the Western diet followed by most Americans [8,9,11,20,21].

The emphasis on plant-rich dietary patterns has contributed to a steep rise in the quantity of plant-based food products available in the US marketplace, particularly plant-based meat alternative (PBMA) products, which aim to mimic the sensory attributes of traditional meat products and undergo substantial industry processing [22,23]. PBMA products may offer environmental and animal welfare benefits compared to traditional meat products [24,25]. However, there is limited evidence that these products will support human health [22,26]. Many PBMA products lack the daily requirements of certain nutrients of which animal-sourced foods are key sources (e.g., vitamin B12 and iron), raising concerns about their use as direct replacements for red meat and other animal-sourced proteins [22,26].

The published literature provides limited insights on American adults’ perceptions, beliefs, and behaviors regarding adopting plant-rich dietary patterns and practices, which could inform community- and population-level strategies to drive greater sustainability action. A 2023 scoping review on sustainable diet-related consumer attitudes and behaviors found only three published US-based studies and four multi-country studies that included US consumers [27]. Research into consumer perceptions and behaviors has mainly focused on the health and environmental aspects of sustainability rather than the use of a multidimensional approach that considers all four sustainability domains (i.e., human health, environmental, social, and economic domains) [27,28]. Encouraging the adoption of plant-rich dietary patterns requires greater understanding of Americans’ beliefs, motivations, and behaviors for selecting food and beverage products that align with such patterns.

The National Health and Nutrition Examination Surveys (NHANES) are the US Government’s primary method for assessing trends in American adults’ health and nutritional status [29], but these surveys do not currently include sustainable diet metrics. Since 2006, the International Food Information Council (IFIC) has independently conducted annual Food and Health Surveys that assess Americans’ perceptions, beliefs, and behaviors related to food and beverage purchases and consumption, including sustainability components. Several publications have described diet and health trends based on the IFIC Food and Health surveys [30,31,32]. However, no published analysis has comprehensively examined the sustainable diet metrics included in these surveys over the past decade (2012–2022).

The purpose of this study is to conduct a secondary data analysis of the annual IFIC Food and Health Surveys carried out over the past decade (2012–2022). We sought to analyze US adults’ perceptions, beliefs, and behaviors regarding plant-rich dietary patterns and practices (e.g., reducing RPM intake and purchasing sustainable food and beverage products) across four sustainability domains (i.e., human health, environmental, social, and economic domains). Differences in perceptions, beliefs, and behaviors based on the respondent’s age, gender, and household income level were assessed to identify how different US adult sub-populations may contribute to sustainable dietary transitions.

## 2. Materials and Methods

### 2.1. IFIC Survey Participants and Data Collection Procedures

The 2012–2022 IFIC Food and Health Surveys were based on a representative sample of the US adult population (aged 18 to 80 years old) and ranged from 62 to 84 survey items each year. Weighting was used to ensure that the distribution of the sample reflected that of the US adult population, guided by the latest annual Current Population Survey available at the time of survey administration [33]. The samples were specifically weighted by age, education, race/ethnicity, region, and gender. The surveys were collected online by Greenwald and Associates using Dynata’s (formerly ResearchNow) consumer panel [34]. The IFIC Food and Health Surveys (2012 to 2022) were completed in an average of 24.2 min. The survey sample size ranged from a high of 1058 respondents in 2012 to a low of 1002 respondents in 2017, with an average sample size of 1012 adult participants per survey year. While select questions remained consistent across the survey years, the IFIC changed some survey questions or response options each year. The full survey results by year are available on the IFIC’s website.

### 2.2. Procedures

For this secondary retrospective study, Food and Health Survey data were obtained through a formal request to the IFIC, after which the researchers independently analyzed the data. Eleven Food and Health Surveys (2012–2022) were reviewed to identify and analyze questions relevant to at least one of the four sustainable diet domains (i.e., human health, environmental, social, and economic domains) [4,5]. Relevant questions were those that were asked in at least two Food and Health Surveys. Open-ended questions, as well as responses that were not offered in at least two survey years, were excluded. Questions about food access, food safety, food waste, and access to nutrition information were beyond the scope of these surveys and, therefore, not analyzed in this study.

### 2.3. Analysis

A cross-tabulation analysis [35] using Statistical Package for the Social Sciences (SPSS) version 29.0 [36] was conducted for 15 IFIC Food and Health Survey questions, as shown in Supplementary Table S1. One question related solely to the health domain, five to the environmental domain, seven to the health and environmental domains, one to the social and environmental domains, and one to all four sustainability domains. Responses to the 15 survey questions were analyzed by household income group to indirectly capture components of the economic sustainability domain. All 15 questions were analyzed by respondents’ age (categorized by generation, based on Pew Research Center definitions [37]), gender, and household income level (Table 1). Questions were analyzed as single items rather than summed or indexed. Survey responses were provided as means and ranges or percentages. Chi-square analyses were conducted on selected questions to assess the significance of trends across survey years. Given the changes in the annual IFIC survey questions and response options over time, more detailed statistical analyses were not feasible for some of the questions.

## 3. Results

This section presents selected results from the cross-tabulation analysis of the 15 IFIC Food and Health survey questions relevant to plant-rich dietary patterns and principles. One question related to the adoption of or adherence to plant-rich dietary patterns. The other 14 relevant IFIC questions related to consumer perceptions, beliefs, and behaviors regarding plant-rich dietary principles. These questions fell into four main categories: red meat and plant protein consumption, sustainable food and beverage considerations, sustainable food and beverage purchases, and access to sustainable food and beverage information.

### 3.1. Plant-Rich Dietary Pattern Adherence

From 2019 to 2022, the percentage of respondents who reported following any plant-rich dietary pattern (i.e., vegetarian, vegan, Mediterranean, plant-based, flexitarian, or Dietary Approaches to Stop Hypertension [DASH]) in the past year more than doubled (12.1% [2019] to 25.8% [2022], *X*^2^[1, *N* = 2060] = 62.788, *p* < 0.001). Younger consumers (i.e., Generation Z [born 1997–2012] and Millennials [born 1981–1996]) were more likely to follow a plant-rich pattern than older consumers (i.e., Generation X [born 1965–1980], Baby Boomers [born 1946–1964], and the Silent Generation [born 1928–1945] [37]) across all four years surveyed. In 2022, nearly twice as many young consumers reported following plant-rich dietary patterns compared to 2019 (19.3% vs. 37.1%, *X*^2^[1, *N* = 748] = 26.894, *p* < 0.001), while more than three times as many older consumers followed these dietary patterns (9.0% vs. 17.3%, *X*^2^[1, *N* = 1312] = 20.307, *p* < 0.001).

Over this time period, vegetarian or vegan dietary pattern adherence varied between 3.1% (2021) and 6.1% (2022), with an average of 4.6% of respondents following vegetarian or vegan dietary patterns. Mediterranean dietary pattern adherence ranged from 4.3% (2021) to 5.8% (2020), with an average of 5.2% of respondents following this dietary pattern, while DASH adherence was steady from 2019 to 2021 (1.9% average), before increasing to 5.5% in 2022. Adherence to a flexitarian dietary pattern significantly increased across the survey years from 2.1% to 7.4% (2019–2022, *X*^2^[1, *N* = 2060] = 32.424, *p* < 0.001), while plant-based dietary adherence increased from 4.0% to 11.8% (*X*^2^[1, *N* = 2060] = 43.624, *p* < 0.001) in the same time period. Younger respondents were more likely to follow plant-based and flexitarian dietary patterns than older generations in all four survey years.

### 3.2. Red Meat and Plant Protein Consumption

In 2016, the IFIC Food and Health Surveys asked consumers about their perceptions of the healthfulness of animal proteins and proteins from plant sources (i.e., “plant proteins”) and added questions about the consumption of these products from 2019. Across all years surveyed (2016–2020), less than half of consumers (38.7% average) indicated that animal proteins were healthy (range: 34.9–42.8%), compared to more than two-thirds (73.5% average) that identified plant proteins as healthy (range: 69.4–75.4%). From 2016 to 2019, the percentage of consumers who stated that animal proteins were unhealthy increased from 10.3% to 16.4%, before dipping to 15.4% in 2020.

The consumption of animal and plant protein products was mixed across respondents. About one-quarter (24.1% average; 2019–2020) of respondents reported that they actively tried to consume animal proteins, while another one-quarter (23.3% average) tried to limit or avoid them; more than one-third of respondents (38.1% average) actively tried to consume plant proteins.

**Figure 2:**
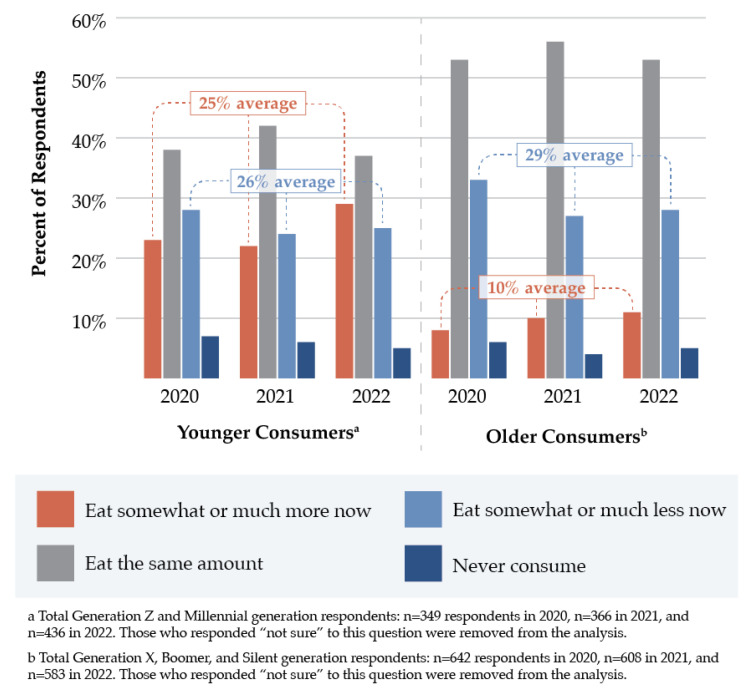
Changes in red meat consumption in the past 12 months among younger versus older consumers, as captured in the International Food Information Council Food and Health Surveys (2020–2022).

From 2020 to 2022, the percentage of respondents who reported eating somewhat or much more red meat in the past 12 months increased from 13.1% to 18.7% (*X*^2^[1, *N* = 2059] = 12.248, *p* < 0.001). Red meat consumption increased over time among both women and men. In 2020, respondents were more than twice as likely to report decreased (31.3%) versus increased (13.1%) red meat intake. However, by 2022, this gap substantially decreased, as the percentage of respondents who reported less red meat intake dropped to 27.1% compared to 18.7% who reported eating more red meat. Women were more likely than men to report reduced red meat intake across all survey years. Younger consumers were substantially more likely to report increased red meat consumption in the past 12 months (25% average) compared to older adults (10% average) in all three survey years (Figure 2). In 2020 and 2021, individuals with lower incomes were more likely to report eating more red meat in the past 12 months than those with higher incomes (15.5% vs. 12.4% average). However, in 2022, more individuals with higher incomes reported increased meat intake (22.3%) compared to those with lower incomes (17.8%).

On average, nearly one fifth (18.8%) of respondents reported increased PBMA intake in the past 12 months (2020–2022). Across the survey years, the number of respondents who reported greater PBMA intake significantly increased (17.0% [2020] to 22.3% [2022], *X*^2^[1, *N* = 2059] = 9.184, *p* < 0.05), particularly among men and individuals from high-income households. In the 2022 survey, the wording for this question was updated from “plant-based meat alternatives” to “plant-based meat and seafood alternatives”, which may have contributed to this trend.

**Figure 3:**
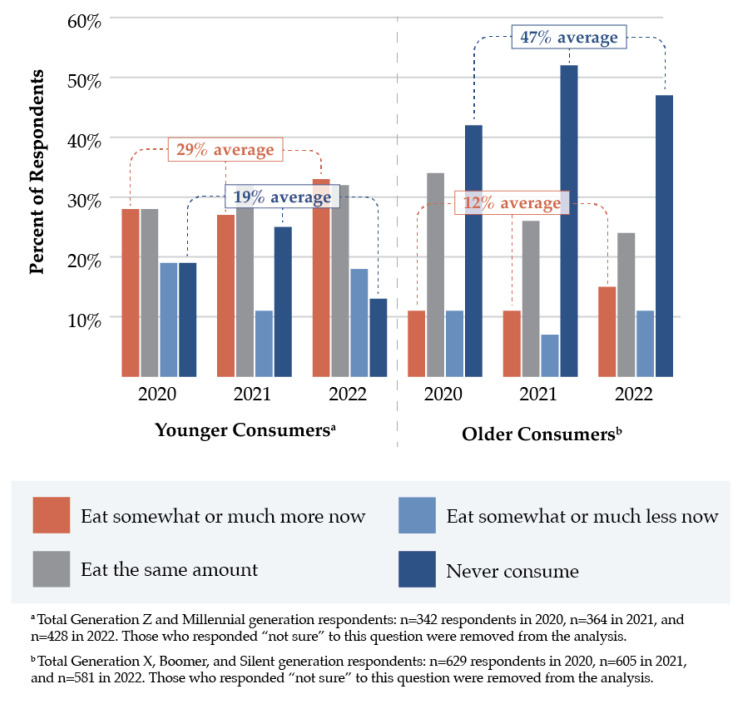
Changes in plant-based meat alternative consumption in the past 12 months between younger and older consumers, as captured in the International Food Information Council Food and Health Surveys (2020–2022).

In 2020, 24.4% of respondents who reported increased PBMA intake also reported increased red meat intake in the previous 12 months. By 2022, 41.0% of those who reported increased PBMA consumption had also increased their red meat intake over the same 12-month period. Younger consumers were more likely than older consumers to report greater PBMA intake (29% vs. 12% average, 2020–2022), while older adults were substantially more likely to report that they never consumed PBMAs (Figure 3). From 2020 to 2021, 12-month PBMA intake was consistent between higher-income and lower-income groups and increased among both groups in 2022, but especially among those with higher household incomes.

### 3.3. Sustainable Food and Beverage Purchases

**Figure 4:**
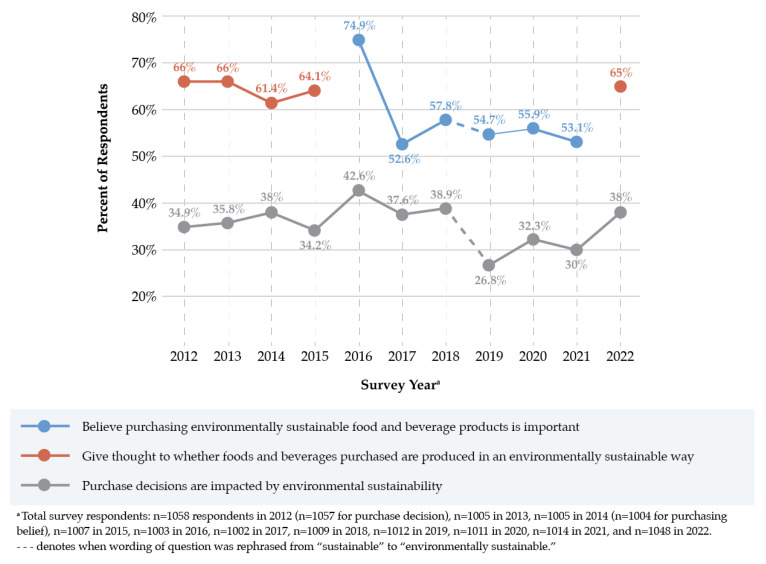
Consumer perceptions, beliefs, and behaviors towards the environmental sustainability impacts of food and beverage products, as captured in the International Food Information Council (IFIC) Food and Health Surveys (2012–2022).

From 2012 to 2018, sustainability was consistently ranked as the least important factor in US consumers’ food and beverage purchase decisions following convenience, price, healthfulness, and taste. On average, only one third of consumers (37.4%) believed that sustainability had a substantial or great influence on their purchase decisions (range: 34.2–42.6%, 2012–2018). In the 2019–2022 surveys, “sustainability” was re-phrased to “environmental sustainability”. Following this change, average consumer responses further declined to less than one third (31.8%) of respondents who indicated that environmental sustainability had a substantial or great impact on purchase decisions (range: 26.8–38.0%, 2019-2022) (Figure 4). Taste was the most important factor for consumers across all years (2012–2022). Women were consistently more likely than men to report that “sustainability” or “environmental sustainability” influenced their purchases over the 11-year survey period.

In comparison, over the past decade, more than 60% of US consumers, on average, indicated that healthfulness had a substantial influence on their food and beverage purchases (range: 59.4–70.7%, 2012–2022). However, between 2012 and 2015, the percentage of consumers who reported giving a lot of thought to the healthfulness of their food and beverage consumption steadily decreased from 57.8% to 48%, and this figure further decreased to 39.6% of consumers in 2022.

Most consumers, particularly women, considered whether food and beverage products were produced in an environmentally sustainable way, and this figure was steady across the survey years (64.4% average, 2012–2015). In 2022, the responses remained consistent, with 65% of respondents indicating that they gave at least a little thought to sustainability, 24.1% of whom gave a lot of thought to this issue (Figure 4). Yet, only half of respondents (50.8%) reported a belief that their individual food and beverage purchases had a moderate or significant impact on the environment, up from 42.5% in 2021. Younger generations were more likely to state that their dietary choices had a significant environmental impact, which decreased with increasing generational age (2021–2022).

When explicitly asked whether purchasing or consuming sustainably produced products was important, 74.9% of respondents reported that it was somewhat or very important in 2016. This value decreased in 2017 but remained consistent between 52.6% and 57.8% each year (2017–2021), even when “sustainable” was rephrased to “environmentally sustainable” for the 2019–2021 surveys (Figure 4). Women were more likely than men to report that purchasing or consuming sustainable products was important to them in each of the years surveyed.

Among consumers who identified sustainable production as being important, conserving natural habitat and reducing pesticide use were the most important sustainable food production aspects identified in all three of the years surveyed (2016–2018). Baby Boomers and Silent Generation respondents were more likely than younger generations to identify pesticide use as an important sustainability concern. Most consumers also indicated that knowing where their food came from influenced their decision to purchase certain foods and beverages (range: 52.7–56.2%, 2017–2020). In 2021 and 2022, consumers were also asked about the significance of knowing that food system workers were treated in a fair and equitable way on food purchases. More than half of respondents (59%) identified this as important in 2021, although this number decreased to 44.7% of respondents in 2022.

### 3.4. Access to Sustainable Food and Beverage Information

On average, nearly two-thirds (64.1%) of respondents, especially women, agreed that it was hard for consumers to know whether the food choices that they made were environmentally sustainable (2019–2020). Most respondents (61.2% average) also agreed that if it was easier to find this information, it would have had a greater influence on their food and beverage choices (2019–2021). Across the three years studied, younger respondents were more likely to agree with this statement than older adults. Less than one quarter of respondents indicated that they purposefully purchased foods labeled as organic (26.5% average, 2019–2021), locally sourced (25.7% average, 2019–2021), environmentally friendly (21% average, 2019–2020), or plant-based (15.5% average, 2020–2021).

Among individuals that identified environmentally sustainable food production methods as important (2019–2022), the most frequent identifiers of sustainably produced product were recyclable packaging and labels that indicated locally grown, sustainably sourced, and non-genetically modified organisms (GMO)/organisms that were not bioengineered (Table 2). Consumers’ reported use of locally grown and organic labels to identify environmentally sustainable food products showed a consistent decline across the survey years, falling from 51.8% (2019) to 32.7% (2022) for locally grown labeling and from 43.4% (2019) to 30.7% (2022) for organic labeling (Table 2).

When asked to compare two products with the same Nutrition Facts Panel, about one-third of consumers believed that a product labeled as “having a small carbon footprint” (32.7% average, 2021–2022) or one that was produced in an environmentally sustainable way (38.1% average, 2019–2021) was healthier than an otherwise identical, non-sustainable product. For both characteristics, younger consumers were more likely than older generations to identify these products as healthier for people and the planet. Similarly, more than 40% of consumers believed that a food labeled as “plant-based” was healthier than an identical alternative product (43.7% average, 2019–2021).

## 4. Discussion

The IFIC survey analysis is discussed in relation to other US-focused consumer research and in the context of future research to better understand consumers’ sustainability-related perceptions, beliefs, and behaviors. Policy and practice recommendations are provided to enhance the enabling environment for consumer behavior change with regard to plant-rich dietary patterns and practices.

### 4.1. Plant-Rich Dietary Pattern Adherence

The IFIC survey results indicate Americans’ low overall adherence to plant-rich dietary patterns, although adherence significantly increased across the survey years and within specific populations. The finding that a small proportion of survey respondents followed a vegetarian or vegan dietary pattern aligns with other US surveys that indicated the low uptake of these patterns. Cramer et al. (2017) [38] found that an estimated 4% of Americans had previously followed a vegetarian or vegan diet for health reasons, with 1.9% having done so within the prior 12 months. Consumer surveys by Gallup (2018) and McKinsey and Company (2022) indicated that 5–8% of Americans surveyed followed a vegetarian or vegan diet [39,40].

Low adoption of plant-rich dietary patterns may be due to a lack of consumer awareness of these dietary patterns or knowledge of their health benefits. For instance, when the flexitarian dietary pattern was described in detail in a 2022 McKinsey and Company consumer survey, 46% of consumers reported following this eating pattern (which was also referred to as “casual vegetarianism”) [39]. A taste preference for meat versus plant-based proteins and disparities in food access and agency (the ability to secure and prepare foods and meals based on one’s context) could also play roles in the low adoption of plant-rich dietary patterns among Americans [27,41].

These findings indicate a need for the provision of greater consumer education by nutrition and health professionals regarding the health, environmental, and animal welfare benefits of adopting such patterns. Educational or social change campaigns like Veganuary [42] and the Have a Plant movement [10], which encourage reducing RPM intake and its replacement with minimally processed plant-based foods, could be used to increase consumer sustainability education and behavior change. While vegetarian dietary patterns that eliminate many or all animal-sourced products generally have the lowest environmental impact [7,8], these patterns may not be acceptable to many Americans. Coordinated efforts are needed to motivate consumers to adopt flexitarian plant-rich dietary patterns (Figure 1) to support both human and planetary health.

### 4.2. Red Meat and Plant Protein Consumption

Studies from the US and other high-income countries have identified low consumer awareness of the environmental impacts of meat production and consumption and low willingness to reduce meat consumption [27,43,44]. Similar to the IFIC Food and Health Survey findings, other recent studies and US consumer polls have indicated that Americans are willing to reduce their meat intake for human health benefits more often than for environmental or animal welfare concerns [27,39,45]. US men, particularly those from low-income households, had higher total and red meat consumption than women and were less likely to reduce meat consumption [45,46,47]. Greater red meat intake among men has been linked to cultural associations between red meat and masculinity [46,47], which may have contributed to the differences seen between genders in IFIC Surveys.

However, the IFIC Food and Health Survey data showed that a significant portion of consumers increased their red meat consumption in recent years, which aligns with analyses showing that per capita red meat consumption has increased in the US over the past decade [48,49]. The Coronavirus Disease of 2019 (COVID-19) pandemic led to decreased red meat production due to facility closures, workforce shortages, and increased sanitation and cleaning procedures, which subsequently contributed to increased red meat prices [50]. The finding that the percentage of Americans who reported greater red meat intake increased amid the COVID-19 pandemic (2020–2022) was, therefore, unexpected.

The IFIC Food and Health Survey results indicated consumer interest in plant-based proteins, coupled with increased consumption of PBMAs, in recent years. A separate 2022 IFIC survey on PBMA consumption found that 65% of Americans had consumed PBMAs in the past year, with healthfulness and high-quality protein cited as the two top reasons for consuming these products [51]. The finding that young consumers reported greater PBMA intake than older adults aligns with prior studies that found that individuals who consumed PBMA products were more likely to be less than 35 years of age [52,53] and live in high-income households (>$100,000) [53]. However, these studies also found that PBMA consumers were more likely to be female [52,53], which was not supported by the IFIC Food and Health Survey findings. The finding that many respondents reported increased red meat and PBMA consumption within the same year supports the evidence suggesting that consumers may eat PBMA products in addition, to rather than in place of, traditional meat products [39,53].

The higher price of PBMA products compared to traditional meat products may have contributed to the smaller increase in PBMA consumption among individuals with lower compared to higher household incomes reported in the 2022 Food and Health Survey [54]. Likewise, nutrition insecurity and participation in federal nutrition assistance programs increased during the COVID-19 pandemic [55], showing that a large proportion of the US population faced financial burdens that may have been reflected in the differences in increased PBMA and red meat consumption between household income groups. Yet, given the increased meat prices during the COVID-19 pandemic [50], it was surprising to find that in the 2022 survey, both income groups reported increased red meat intake in the past 12 months (a reflection of their 2021–2022 intake). Government food assistance initiatives (e.g., the Farmers to Families Food Box initiative and the Emergency Food Assistance Program) that, during the COVID-19 pandemic, distributed US agricultural products, including meats, to individuals and families in need may have contributed to this increase [56]. The 2022 finding that individuals with lower incomes were less likely to report increased red meat intake aligns with findings from Ritzel & Mann (2021) [46]. These researchers found that household income has a negative effect on total and red meat consumption in the US [46]. However, the results of the 2020 and 2021 IFIC Surveys did not support this trend. Future research should study how income differences affect US consumers’ willingness and capacity to reduce red meat intake and increase consumption of plant-based proteins.

Increased action is needed to nudge American consumers towards sustainable dietary changes. Taxes on high-environmental-impact products like RPM, subsidies for low-impact products, or making sustainable food choices the default at food outlets could be used to shift consumer behavior to support planetary health [27,44]. Adopting subsidies for healthy foods (i.e., fruits, vegetables, and whole grains) has the potential to reduce NCD deaths and improve food access and economic health disparities in the US [57]. Given concerns about the implications of highly processed PBMA products for human health, places, and the planet [26], additional research and US Government and expert guidance is needed to explain how consumers should incorporate these products into plant-rich dietary patterns.

### 4.3. Sustainable Food and Beverage Purchases

The findings of IFIC Food and Health Surveys indicate a discrepancy between consumers’ perceptions of environmentally sustainable food and beverage products and their behaviors. Although most adult consumers reported thinking about whether the products that they purchased were produced in an environmentally sustainable way and supported purchasing sustainable products, these thoughts did not appear to influence many consumers’ purchasing decisions. A 2022 McKinsey and Company consumer survey also found that while most US consumers (64%) identified sustainable solutions as important, less than half of these consumers reported a willingness to pay more for sustainable options or eat more sustainable products [39].

The past decade of IFIC Food and Health Surveys showed that US consumers ranked environmental sustainability far below many other metrics (i.e., convenience, healthfulness, price, and taste) that influenced their food and beverage purchase decisions. Similarly, a 2022 multi-country systematic review found that taste, price, and individual health influenced consumers’ food purchases more than environmental sustainability [43]. A 2022 assessment of how American consumers’ shopping habits aligned with healthy diets for sustainable food systems found that taste and economic factors, such as affordability and access, ranked above environmental and social factors [58]. These results suggest that despite being interested in supporting environmentally and socially sustainable products, consumers either purchased fewer of these products or were unaware of how these products may support sustainable diets. The 2023 IFIC Food and Health Survey revealed that most Americans were not willing to pay more for healthier or socially sustainable products due to inflation and the consequent rising prices of groceries [59].

The decrease in Food and Health Survey respondents’ recognition of the importance of fair and equitable worker treatment between 2021 and 2022 may have been a result of the COVID-19 pandemic. Vulnerabilities in the US food system and inequities and injustice among food system workers deemed essential during the pandemic were at the forefront of media coverage in 2020–2021 period [60,61].

Notably absent from the IFIC Food and Health Surveys were questions that addressed economic sustainability principles, such as the impact of food accessibility and affordability on consumers’ sustainability actions. Shifting towards more sustainable diets that support human and planetary health may not be affordable for low-income Americans [62]. This highlights the need to consider the economic sustainability domain when assessing consumer behaviors and their policy implications [5]. Current research into sustainable dietary patterns emphasizes the environmental and health benefits of such diets [4,27], but additional research is needed to fully capture their economic and social implications for Americans.

### 4.4. Access to Sustainable Food and Beverage Information

The IFIC Food and Health Survey findings indicate that consumers are interested in but cannot access environmental sustainability information about the food and beverage products that they purchase and consume. Similarly, a 2022 McKinsey and Company consumer survey identified that nearly half of US consumers had trouble understanding what actions to take to be more sustainable [39]. The IFIC Survey results also indicate that consumers having a clearer understanding of how their dietary behaviors could influence sustainability and drive greater adoption of plant-rich dietary practices. Consumers need greater transparency from industry to access environmental and social sustainability information. Advocates and civil-society organizations should hold industry accountable for prioritizing the influence of their food and beverage products across all four sustainability domains, as well as for publicizing this information through the annual reporting of sustainability metrics.

Many US companies are adopting labeling schemes that highlight the ecological or social sustainability aspects of their products, such as carbon footprint labels [63]. These labeling schemes are voluntary and largely led by industry. The IFIC Survey results indicate that consumers may misconstrue products with environmental sustainability attributes (i.e., small carbon footprint labels, sustainable production methods) as being implicitly healthier, creating a “health halo” around these products. Research indicates that nutrition labeling can encourage consumers to make healthy choices and push industry to reformulate products [64]. Yet, the complexity around capturing a product’s impacts across the four sustainability domains and the lack of unified, independent certification schemes and regulatory oversight raises concerns about the future of sustainability labeling by industry and political actors [65]. Additional research is needed regarding the impact of the US food and beverage industry’s sustainability labels and product attributes on consumers’ perceptions and behaviors.

The finding that consumers’ use of locally grown and organic labels to identify environmentally sustainable products decreased across years (2019–2022) is unexpected, as consumer demand for local and organic products has grown over the past decade [66,67]. However, this result aligns with the finding that only one-quarter of all respondents, on average, purposefully purchased organic or locally sourced food and beverage products. Consumers’ use of all environmental sustainability food labeling characteristics declined between 2019 and 2022 (Table 2), which could indicate growing consumer confusion or distrust of food labels. There may be growing consumer awareness of the fraud or greenwashing that has historically surrounded organic labeling [68], as well as the lack of government oversight for many common food labels [69].

US government agencies are in the process of updating guidelines for sustainability and eco-labeling claims [70,71]. Therefore, nutrition and health professionals, advocates, and civil society should encourage the food and beverage industry to adopt independent sustainability labeling schemes based on clearly defined criteria certified by third-party organizations, such as the Cool Food Initiative adopted by Panera restaurant in partnership with the World Resources Institute [72]. These efforts are needed to help consumers to navigate through the abundant and unregulated labeling used to market food and beverage products to identify those that support sustainability. However, research indicates that given the sociocultural and economic barriers to accessing healthy and sustainable food and beverage products, coupled with the low cost of many meat products, further information alone will not drive consumers to adopt more sustainable behaviors [27,73]. Sustainability labeling and education campaigns should be used in coordination with more intrusive policy tools, such as taxes or food subsidies, to effectively drive behavioral change [73].

### 4.5. Strengths and Limitations and Recommendations for Future IFIC Food and Health Surveys

The strength of this study is the novel analysis of IFIC Food and Health Survey questions over a decade from a representative sample of US adults, providing insights into US consumers’ sustainability-related perceptions, beliefs, and behaviors. The limitations of this study largely stem from the design of the Food and Health Surveys. The cross-sectional nature of the surveys limits researchers’ capacity to analyze changes in individual consumers’ perceptions, beliefs, and behaviors over time. Changes in the variety of questions asked and response options provided from year to year further limited our capacity to analyze trends and conduct more in-depth statistical analyses for certain questions. Across the decade of IFIC Surveys, many changes were made to the wording of certain questions and the response options provided. The authors have clarified where these changes occurred throughout this paper, as detailed in Supplementary Table S1. These changes may have impacted the results of this analysis.

The IFIC Surveys captured participant age data on a continuous scale, which the authors categorized into generations for the purpose of this analysis. The limitations in the representation of certain generational groups (i.e., low frequencies of Generation Z respondents in earlier survey years) may have impacted the presence or absence of generational trends observed across the surveys. While the IFIC Survey results are weighted to reflect certain characteristics of the US population, the responses captured from the ~1000 survey respondents in each survey year may not be reflective of the perceptions, beliefs, and behaviors of the entire US population. Some results presented in this survey, therefore, may have occurred due to the differences in the individuals sampled across survey years. The administration of the survey online also likely excluded those with low digital literacy skills and those who lacked access to the internet or digital technologies.

The IFIC Survey questions are also not based on a publicly reported conceptual framework for health behavioral change, which further limited our capacity to analyze certain cognitive and behavioral outcomes related to sustainability. Future Food and Health Surveys should standardize sustainability questions to allow for greater analysis of trends in consumer responses over time. IFIC should also consider utilizing a research-grounded conceptual framework for behavioral change to guide the formation of Food and Health Survey questions. Future IFIC Surveys should use standardized sustainable diet-relevant questions, so that the IFIC Surveys could serve as a reliable method for capturing ongoing sustainable diet metrics for US adults.

In addition to supporting human and environmental health, sustainable diets and food systems must also advance social equity and economic prosperity for current and future generations [4,6]. Future IFIC Food and Health Surveys should, therefore, include additional questions that address economic and social sustainability domains that have been under-represented in these surveys over the past decade. Additional consideration should be given to developing questions that can help to better assess Americans’ perceptions of and interest in reducing RPM intake and adopting sustainable dietary principles in line with expert recommendations, such as the frequency, types, and portion sizes of animal and plant protein intake by Americans.

In the 2012 to 2018 IFIC Food and Health Surveys, “male” (described as “men”) and “female” (described as “women”) were the only two gender options from which survey participants could choose. In 2019, “other” and “prefer not to say” were added as options but were not included in this analysis (Table 1). Future IFIC surveys should incorporate additional gender options to be more inclusive of and better capture the breadth of the gender identities of US adults. Moreover, IFIC Survey findings may not be applicable to other high-income countries or to US children or adolescents.

## 5. Conclusions

The IFIC Food and Health Survey findings from the past decade indicate US consumer awareness of and interest in health, environmental, and social sustainability principles but low adoption of plant-rich dietary patterns or practices, especially to reduce RPM intake. Greater action is needed by health and nutrition professionals, industry, civil society, and governments to educate and nudge consumers to adopt plant-rich dietary patterns and reduce or replace RPM intake to support the health of people and the planet.

## Figures and Tables

**Table 1:** Demographic characteristics of respondents used for the International Food Information Council Food and Health Survey analysis (2012–2022).

Demographic Characteristic	Groups Used for Analysis	Source
Gender	Male, female	2012–2018 Food and Health Surveys: “male” and “female” were the only gender options that respondents could choose. 2019–2022 Food and Health Surveys: “prefer not to say” and “other” response options were added. ^1^
Generation (years)	Silent Generation (born 1928–1945) Baby Boomers (born 1946–1964) Generation X (born 1965–1980) Millennials (born 1981–1996) Generation Z (born 1997–2012)	2012–2022 Food and Health Surveys: age was collected as a continuous variable (18–80 years old). Generational age based on Pew Research Center definitions [37].
Household income	Lower income ($0 to <$75,000 USD) Higher income ($75,000 to $150,000+ USD)	The 2012–2022 Food and Health Surveys included six income groups that were divided into two groups (i.e., lower income and higher income) for this analysis. ^2^

USD = US dollars. ^1^ Only “male” and “female” options were analyzed for consistency across survey years, and they are referred to as “men” and “women”, respectively, in this paper. ^2^ Individuals who responded “not sure” or “prefer not to answer” for household income level were excluded from the income-related analyses.

**Table 2:** Food product characteristics that consumers reported using to identify environmentally sustainable food products in the International Food Information Council Food and Health Surveys (2019–2022).

	Survey Year (*n* = Number of Respondents ^1^)				
Response Option	2019 (*n* = 554)	2020 (*n* = 565)	2021 (*n* = 538)	2022 (*n* = 784)	2019–2022 Average
Recyclable Packaging	43.9%	45.5%	53.0%	40.8%	45.8%
Minimal/Reusable Packaging	37.0%	35.6%	42.4%	29.6%	36.2%
Labeled as Organic	43.3%	40.4%	34.8%	30.7%	37.3%
Labeled as Being Locally Grown	51.8%	43.4%	38.1%	32.7%	41.5%
Labeled as Sustainably Sourced	46.0%	47.1%	45.2%	35.1%	43.4%
Labeled as Non-GMO/Not Bioengineered	46.2%	42.7%	35.7%	27.8%	38.1%
Labeled as Bioengineered/ Containing Bioengineered Ingredients ^2^	N/A	17.0%	13.2%	16.5%	15.6%

GMO = Genetically modified organism. ^1^ This question was only asked to individuals who responded that it was “somewhat important” or “very important” to know that the products that they purchase or consume are produced in a sustainable way, so these totals do not align with the total survey participants for 2019–2022. ^2^ This response option was only offered in the 2020–2022 surveys.

## Data Availability

The International Food Information Council (IFIC) Survey data used in this analysis were obtained through a request from the organization. All requests for this data should, therefore, be directed to the IFIC.

## Supplementary Materials

The following supporting information can be downloaded via this link: https://www.mdpi.com/article/10.3390/nu15234990/s1, Table S1: International Food Information Council (IFIC) Food and Health Survey questions about perceptions, beliefs, and behaviors towards plant-rich dietary patterns and practices (2012–2022).

## References

[1] Searchinger T., Waite R., Hanson C., Ranganathan J. (2019). Creating a Sustainable Food Future: A Menu of Solutions to Feed Nearly 10 Billion People by 2050.

[2] The Rockefeller Foundation (2021). True Cost of Food: Measuring What Matters to Transform the U.S. Food System. https://www.rockefellerfoundation.org/report/true-cost-of-food-measuring-what-matters-to-transform-the-u-s-food-system/.

[3] Hebinck A., Zurek M., Achterbosch T., Forkman B., Kuijsten A., Kuiper M., Nørrung B., van ’t Veer P., Leip A. (2021). A Sustainability Compass for policy navigation to sustainable food systems. Glob. Food Secur..

[4] Drewnowski A., Finley J., Hess J.M., Ingram J., Miller G., Peters C. (2020). Toward healthy diets from sustainable food systems. Curr. Dev. Nutr..

[5] Kraak V.I., Consavage Stanley K. (2023). An economic lens for sustainable dietary guidelines. Lancet Planet Health.

[6] Food and Agriculture Organization of the United Nations, World Health Organization (2019). Sustainable Healthy Diets: Guiding Principles.

[7] Reinhardt S.L., Boehm R., Blackstone N.T., El-Abbadi N.H., McNally Brandow J.S., Taylor S.F., DeLonge M.S. (2020). Systematic review of dietary patterns and sustainability in the United States. Adv. Nutr..

[8] Jennings R., Henderson A.D., Phelps A., Janda K.M., van den Berg A.E. (2023). Five U.S. dietary patterns and their relationship to land use, water use, and greenhouse gas emissions: Implications for future food security. Nutrients.

[9] Blackstone N.T., El-Abbadi N.H., McCabe M.S., Griffin T.S., Nelson M.E. (2018). Linking sustainability to the healthy eating patterns of the Dietary Guidelines for Americans: A modelling study. Lancet Planet Health.

[10] Produce for Better Health Foundation Have a Plant: The Plant-Forward Eating Guide. n.d. https://fruitsandveggies.org/wp-content/uploads/2021/01/Have-A-Plant-Plant-Forward-Eating-Guide.pdf.

[11] Lichtenstein A.H., Appel L.J., Vadiveloo M., Hu F.B., Kris-Etherton P.M., Rebholz C.M., Sacks F.M., Thorndike A.N., Van Horn L., Wylie-Rosett J. (2021). 2021 Dietary guidance to improve cardiovascular health: A scientific statement from the American Heart Association. Circulation.

[12] EAT-Lancet Commission (2019). Healthy Diets from Sustainable Food Systems. Summary Report of the EAT-Lancet Commission. https://eatforum.org/content/uploads/2019/07/EAT-Lancet_Commission_Summary_Report.pdf.

[13] Dietary Guidelines Advisory Committee (2015). Scientific Report of the 2015 Dietary Guidelines Advisory Committee: Advisory Report to the Secretary of Health and Human Services and the Secretary of Agriculture.

[14] Vega Mejía N., Ponce Reyes R., Martinez Y., Carrasco O., Cerritos R. (2018). Implications of the western diet for agricultural production, health and climate change. Front. Sustain. Food Syst..

[15] Dietary Guidelines Advisory Committee (2020). Scientific Report of the 2020 Dietary Guidelines Advisory Committee: Advisory Report to the Secretary of Agriculture and Secretary of Health and Human Services.

[16] Qian F., Riddle M.C., Wylie-Rosett J., Hu F.B. (2020). Red and processed meats and health risks: How strong is the evidence?. Diabetes Care.

[17] Neuenschwander M., Ballon A., Weber K.S., Norat T., Aune D., Schwingshackl L., Schlesinger S. (2019). Role of diet in type 2 diabetes incidence: Umbrella review of meta-analyses of prospective observational studies. BMJ.

[18] World Cancer Research Fund International, American Institute for Cancer Research (2018). Continuous Update Project. Recommendations and Public Health and Policy Implications. https://www.wcrf.org/wp-content/uploads/2021/01/Recommendations.pdf.

[19] Poore J., Nemecek T. (2018). Reducing food’s environmental impacts through producers and consumers. Science.

[20] Carey C.N., Paquette M., Sahye-Pudaruth S., Dadvar A., Dinh D., Khodabandehlou K., Liang F., Mishra E., Sidhu M., Brown R. (2023). The environmental sustainability of plant-based dietary patterns: A scoping review. J. Nutr..

[21] Dinu M., Abbate R., Gensini G.F., Casini A., Sofi F. (2017). Vegetarian, vegan diets and multiple health outcomes: A systematic review with meta-analysis of observational studies. Crit. Rev. Food Sci. Nutr..

[22] Toh D.W.K., Srv A., Henry C.J. (2022). Unknown impacts of plant-based meat alternatives on long-term health. Nat. Food.

[23] Ismail I., Hwang Y.H., Joo S.T. (2020). Meat analog as future food: A review. J. Anim. Sci. Technol..

[24] Heller M.C., Keoleian G.A. (2018). Beyond Meat’s Beyond Burger Life Cycle Assessment: A Detailed Comparison Between a Plant-Based and an Animal-Based Protein Source. University of Michigan Center for Sustainable Systems. https://gastronomiaycia.republica.com/wp-content/uploads/2018/09/diferencias_hamburguesa_vegetal_carne_impacto_medioambiental.pdf.

[25] The Good Food Institute (2019). Plant-based Meat for a Growing World. https://gfi.org/wp-content/uploads/2021/02/GFI-Plant-Based-Meat-Fact-Sheet_Environmental-Comparison.pdf.

[26] Prescott S.L., D’Adamo C.R., Holton K.F., Ortiz S., Overby N., Logan A.C. (2023). Beyond plants: The ultra-processing of global diets is harming the health of people, places, and planet. Int. J. Environ. Res. Public Health.

[27] Kenny T.A., Woodside J.V., Perry I.J., Harrington J.M. (2023). Consumer attitudes and behaviors toward more sustainable diets: A scoping review. Nutr. Rev..

[28] Miki A.J., Livingston K.A., Karlsen M.C., Folta S.C., McKeown N.M. (2020). Using evidence mapping to examine motivations for following plant-based diets. Curr. Dev. Nutr..

[29] Centers for Disease Control and Prevention, National Center for Health Statistics About the National Health and Nutrition Examination Survey.pdf. Updated 31 May 2023. https://www.cdc.gov/nchs/nhanes/about_nhanes.htm.

[30] Sollid K., Webster A.D., Paipongna M., Smith K. (2022). Food perceptions, beliefs, and behaviors amid a global pandemic: Results of the International Food Information Council 2021 Food & Health Survey. Nutr. Today.

[31] Hornick B.A., Childs N.A., Smith Edge M., Reinhardt K.W., Dooher C., White C. (2012). Is it time to rethink nutrition communications? A 5-year retrospective of Americans’ attitudes toward food, nutrition, and health. J. Acad. Nutr. Diet..

[32] Goldberg J.P., Tanskey L.A., Sanders E.A., Smith Edge M. (2016). The IFIC Foundation Food & Health Survey 2015: 10-year trends and emerging issues. J. Acad. Nutr. Diet..

[33] Food Insight (2021). 2021 IFIC Food & Health Survey. International Food Information Council. https://foodinsight.org/2021-food-health-survey/.

[34] Food Insight (2022). 2022 Food & Health Survey. International Food Information Council. https://foodinsight.org/2022-food-and-health-survey/.

[35] Carpenter A. Cross-Tabulation Analysis: A Researcher’s Guide. Qualtrics, n.d. https://www.qualtrics.com/experience-management/research/cross-tabulation/.

[36] IBM IBM SPSS Statistics Documentation. n.d. https://www.ibm.com/docs/en/spss-statistics.

[37] Dimock M. (2019). Defining Generations: Where Millennials End and Generation Z Begins. Pew Research Center. https://www.pewresearch.org/short-reads/2019/01/17/where-millennials-end-and-generation-z-begins/.

[38] Cramer H., Kessler C.S., Sundberg T., Leach M.J., Schumann D., Adams J., Lauche R. (2017). Characteristics of Americans choosing vegetarian and vegan diets for health reasons. J. Nutr. Educ. Behav..

[39] Grimmelt A., Moulton J., Pandya C., Snezhkova N. (2022). Hungry and Confused: The Winding Road to Conscious Eating. McKinsey Company. https://www.mckinsey.com/industries/consumer-packaged-goods/our-insights/hungry-and-confused-the-winding-road-to-conscious-eating.

[40] Reinhart R. (2018). Snapshot: Few Americans Vegetarian or Vegan. Gallup. https://news.gallup.com/poll/238328/snapshot-few-americans-vegetarian-vegan.aspx.

[41] Wolfson J.A., Lahne J., Raj M., Insolera N., Lavelle F., Dean M. (2020). Food agency in the United States: Associations with cooking behavior and dietary intake. Nutrients.

[42] Veganuary About Us. n.d. https://veganuary.com/about/.

[43] van Bussel L.M., Kuijsten A., Mars M., van‘t Veer P. (2022). Consumers’ perceptions on food-related sustainability: A systematic review. J. Clean Prod..

[44] Hartmann C., Siegrist M. (2017). Consumer perception and behaviour regarding sustainable protein consumption: A systematic review. Trends Food Sci. Technol..

[45] Mccarthy J., Dekoster S. (2020). Nearly One in Four in U.S. Have Cut Back on Eating Meat. Gallup. https://news.gallup.com/poll/282779/nearly-one-four-cut-back-eating-meat.aspx.

[46] Ritzel C., Mann S. (2021). The old man and the meat: On gender differences in meat consumption across stages of human life. Foods.

[47] Nakagawa S., Hart C. (2019). Where’s the beef? How masculinity exacerbates gender disparities in health behaviors. Socius Sociol. Res. Dyn. World.

[48] Economic Research Service, US Department of Agriculture Per Capita Red Meat and Poultry Consumption Expected to Decrease Modestly in 2022. Updated 15 April 2022. https://www.ers.usda.gov/data-products/chart-gallery/gallery/chart-detail/?chartId=103767#:~:text=The%20latest%20USDA%20forecast%20indicates,than%20the%202012-21%20average.

[49] Our World in Data Per Capita Meat Consumption in the United States. n.d. https://ourworldindata.org/grapher/per-capita-meat-usa.

[50] Whitehead D., Brad Kim Y.H. (2022). The Impact of COVID 19 on the Meat Supply Chain in the USA: A Review. Food Sci. Anim. Resour..

[51] International Food Information Council (2021). Consumption Trends, Preferred Names and Perceptions of Plant-Based Meat Alternatives. https://foodinsight.org/wp-content/uploads/2021/11/IFIC-Plant-Based-Meat-Survey.November-2021.pdf.

[52] Onwezen M.C., Bouwman E.P., Reinders M.J., Dagevos H. (2021). A systematic review on consumer acceptance of alternative proteins: Pulses, algae, insects, plant-based meat alternatives, and cultured meat. Appetite.

[53] Neuhofer Z.T., Lusk J.L. (2022). Most plant-based meat alternative buyers also buy meat: An analysis of household demographics, habit formation, and buying behavior among meat alternative buyers. Sci. Rep..

[54] Nezlek J.B., Forestell C.A. (2022). Meat substitutes: Current status, potential benefits, and remaining challenges. Curr. Opin. Food Sci..

[55] Caspi C., Seligman H., Berge J., Ng S.W., Krieger J. (2022). COVID-19 Pandemic-Era Nutrition Assistance: Impact and Sustainability. *Health Affairs*. https://www.healthaffairs.org/do/10.1377/hpb20220330.534478/full/.

[56] Leone L.A., Fleischhacker S., Anderson-Steeves B., Harper K., Winkler M., Racine E., Baquero B., Gittelsohn J. (2020). Healthy food retail during the COVID-19 pandemic: Challenges and future directions. Int. J. Environ. Res. Public Health.

[57] Peñalvo J.L., Cudhea F., Micha R., Rehm C.D., Afshin A., Whitsel L., Wilde P., Gaziano T., Pearson-Stuttard J., O’Flaherty M. (2017). The potential impact of food taxes and subsidies on cardiovascular disease and diabetes burden and disparities in the United States. BMC Med..

[58] Lusk J.L., Polzin S. (2022). Consumer Food Insights. Center for Food Demand Analysis and Sustainability. Purdue University. https://ag.purdue.edu/next-moves/consumer-food-insights/?_ga=2.134760241.1325228140.1645811973-1258680646.1645811973.

[59] Food Insight (2023). 2023 Food & Health Survey. International Food Information Council. https://foodinsight.org/2023-food-and-health-survey/.

[60] Jordan M. (2020). Farmworkers, Mostly Undocumented, Become ‘Essential’ During Pandemic. *The New York Times*. https://www.nytimes.com/2020/04/02/us/coronavirus-undocumented-immigrant-farmworkers-agriculture.html.

[61] Stuesse A. (2020). Coronavirus Can Afflict The Powerful. Yet Food Workers Remain The Most Vulnerable. *The Washington Post*. https://www.washingtonpost.com/outlook/2020/10/04/covid-19-can-afflict-powerful-yet-food-workers-remain-most-vulnerable/.

[62] He P., Feng K., Baiocchi G., Sun L., Hubacek K. (2021). Shifts towards healthy diets in the US can reduce environmental impacts but would be unaffordable for poorer minorities. Nat. Food.

[63] Wolfrom J. (2021). Companies bet carbon labels can help the climate. Will consumers catch on? *The Washington Post*. https://www.washingtonpost.com/climate-solutions/2021/06/17/carbon-footprint-emissions-label/.

[64] Shangguan S., Afshin A., Shulkin M., Ma W., Marsden D., Smith J., Saheb-Kashaf M., Shi P., Micha R., Imamura F. (2019). A meta-analysis of food labeling effects on consumer diet behaviors and industry practices. Am. J. Prev. Med..

[65] Brown K.A., Harris F., Potter C., Knai C. (2020). The future of environmental sustainability labelling on food products. Lancet Planet Health.

[66] Economic Research Center, US Department of Agriculture Organic Agriculture Overview. Updated 13 February 2023. https://www.ers.usda.gov/topics/natural-resources-environment/organic-agriculture/.

[67] Brinkmeyer E., Dankbar H., Bloom J.D. (2023). Local Food Systems: Clarifying Current Research. North Carolina State Extension. https://content.ces.ncsu.edu/local-food-systems-clarifying-current-research.

[68] Reiley L. (2023). USDA Moves to Crack Down on ‘Organic’ Fraud. *The Washington Post*. https://www.washingtonpost.com/business/2023/01/19/usda-rule-organic-fraud/#.

[69] Tufts University Decoding Food Labels. Updated August 2016. https://sustainability.tufts.edu/wp-content/uploads/Decoding-Food-Labels.pdf.

[70] Federal Trade Commission (2022). FTC Seeks Public Comment on Potential Updates to its ‘Green Guides’ for the Use of Environmental Marketing Claims. https://www.ftc.gov/news-events/news/press-releases/2022/12/ftc-seeks-public-comment-potential-updates-its-green-guides-use-environmental-marketing-claims.

[71] US Department of Agriculture (2023). USDA Publishes Strengthening Organic Enforcement Final Rule. https://www.usda.gov/media/press-releases/2023/01/18/usda-publishes-strengthening-organic-enforcement-final-rule.

[72] Waite R., Vennard D., Pozzi G. (2019). Tracking Progress Toward the Cool Food Pledge: Setting Climate Targets, Tracking Metrics, Using the Cool Food Calculator, and Related Guidance for Pledge Signatories, World Resources Institute. https://www.wri.org/research/tracking-progress-toward-cool-food-pledge.

[73] Ammann J., Arbenz A., Mack G., Nemecek T., El Benni N. (2023). A review on policy instruments for sustainable food consumption. Sustain. Prod. Consum..

