# Supplementary material for: US Adults’ Perceptions, Beliefs, and Behaviors towards Plant-Rich Dietary Patterns and Practices: International Food Information Council Food and Health Survey Insights, 2012–2022
Source: Nutrients. 2023 Dec 1;15(23):4990. doi: 10.3390/nu15234990 (PMC10708400; doi:10.3390/nu15234990)
Supplement: Supplementary file 1 [file nutrients-15-04990-s001.zip › nutrients-2719623-supplementary.pdf]

Supplemental Table S1. International Food Information Council Food and Health Survey questions about perceptions, beliefs, and behaviors towards plant-rich dietary patterns and practices (2012-2022).

Survey Question	Response Options	Sustainability Domain(s)	Years Surveyed	Notes
How much of an impact do the following have on your decision to buy foods and beverages?<br>a) Convenience<br>b) Healthfulness<br>c) Price<br>d) Taste<br>e) (Environmental) sustainability	Scale of 1 (no impact) to 5 (a great impact)	Environmental; Health	2012-2022	Option e) listed as "sustainability" in surveys from 2012-2018, then updated to "environmental sustainability" in 2019-2022 surveys<br><br>Response options 1 and 2 (i.e., "no impact" and "little impact") were combined for this analysis, as were options 4 and 5 (i.e., substantial impact" and "great impact")
Which of the following, if any, do you purposefully do on a regular basis? (That is, most times when you shop for foods and beverages)? Buy foods and beverages because they are advertised on the label as… (select all that apply)	• Organic (2012-2022)<br>• Natural (2012-2022)<br>• Locally sourced (2012-2022)<br>• Raised without antibiotics/antibiotic free (2014-2022)<br>• No added hormones or steroids (2014-2022)<br>• Sustainably sourced (2015-2022)<br>• Pesticide-free (2016-2020)<br>• Non-Genetically Modified Organism (GMO) (2017-2022)<br>• Environmentally friendly (2019-2020)<br>• Plant-based (2020-2022)<br>• Made using regenerative agriculture (2020-2021)<br>• Clean ingredients (2020-2022)<br>• None of the above (2012-2022)<br>• Bioengineered/containing bioengineered ingredients (2020-2022)	Environmental; Health	2012-2022	Split sample was done in 2017 with another question (only half of respondents were asked this question).<br><br>Survey question wording was re-structured in 2015 but the underlying concept remained unchanged

	• Fair wage or fair trade (2021-2022)			
Over the past year, how much thought have you given to the following issues?<br>• Whether your foods and beverages are produced in a sustainable way<br>• The healthfulness of the foods and beverages you consume<br>• The ways the foods and beverages you consume are farmed or produced (2014-2015 only)	• A lot<br>• A little<br>• None<br>• Not sure	Environmental; Health	2012-2015; 2022	Other constructs were asked in this question, but they were not directly related to sustainability and therefore not included in this analysis.
How important is it to you that the food products you purchase or consume are produced in a (environmentally) sustainable way?	• Not at all important<br>• Not very important<br>• Neither important or unimportant<br>• Somewhat important<br>• Very important<br>• Don't know enough to form an opinion	Environmental	2016-2021	This question was updated from "sustainable" (used in the 2012-2018 surveys) to "environmentally sustainable" in the 2019-2022 surveys.<br><br>"Not at all important" and "not very important" were combined for the analysis, as were "Somewhat important" and "very important"
What 3 aspects of producing food in a sustainable way are most important to you?	Respondents were asked to select three of the following responses and rank them from 1 (most important) to 3:<br>• Ensuring a sufficient food supply for the growing global population<br>• Conserving the natural habitat (water, land, rainforests, etc.)<br>• Ensuring an affordable food supply<br>• Producing more food with less use of natural resources<br>• Reducing the amount of pesticides used to produce food<br>• Less food and energy waste	All domains (i.e., Health, Environmental, Social, and Economic domains)	2016-2018	This question was only asked to individuals who responded "somewhat important" or "very important" to the question above about how important it is that foods product purchased are sustainable.<br><br>Response options that were only offered in one

	• Few food miles (that is, shorter distance from farm to point of purchase)<br>• Conserving farmland over multiple generations<br>• None of the above are important to you			survey year were not included in this analysis.
How would you rate the healthfulness of each of the following?<br>• Animal protein<br>• Protein from plant sources	• Unhealthy<br>• Neither healthy or unhealthy<br>• Healthy<br>• Not sure	Health	2016-2020	
How important are the following factors in your decision to purchase a food or beverage?<br>• Knowing where the food comes from (2017-2020)<br>• Knowing that the manufacturer has a commitment to producing food in an environmentally sustainable way (2018-2020, 2022)<br>• Knowing that the food was produced with animal welfare in mind (2018-2022)<br>• Being able to access information about how my food is produced (2018-2020)<br>• Knowing whether the food is a bioengineered food or contains bioengineered ingredients (2020-2022)<br>• Knowing that the food was produced using farming technologies that seek to reduce the impact on natural resources (2020-2022)<br>• Knowing that the food manufacturer has a commitment to reducing their carbon footprint (the amount of greenhouse gases generated) (2021-2022)<br>• Knowing that the workers who produce, distribute or serve the food (e.g., farm workers, factory workers, retail and foodservice staff) are treated in a fair and equitable way (2021-2022)<br>• Whether it has recyclable packaging (2017, 2022)	Response options scale of 1 (not at all important) to 5 (very important)	Environmental; Social	2017-2022	In 2021, the fair and equitable worker treatment was asked as a separate question but with the same construct and response options, so it was compared with 021 responses for this question.<br><br>In 2022, a split sample was done for "knowing that the food or beverage is produced in an environmentally sustainable way" and "knowing that the food or beverage is produced in a way that minimizes its carbon footprint/climate impact" (new in 2022) with half of participants asked the importance of each of these factors.<br><br>Response options 1 and 2 were combined for this

				analysis, as were options 4 and 5
Have you followed any specific eating pattern or diet at any time in the past year? Select all that apply.	• Cleanse [SPECIFY]<br>• Intermittent fasting<br>• Paleo diet<br>• High-protein diet [SPECIFY]<br>• Vegetarian or vegan diet [SPECIFY]<br>• Whole 30<br>• Low-carb diet [SPECIFY]<br>• Weight-loss plan [SPECIFY]<br>• Mediterranean diet<br>• Dietary Approaches to Stop Hypertension (DASH) diet<br>• Gluten-free diet<br>• Ketogenic or high-fat diet<br>• Low-fat diet [SPECIFY]<br>• Plant-based diet (2018-2022)<br>• Flexitarian (2018-2022)<br>• Clean eating (2018-2022)<br>• Carb cycling (2020-2022)<br>• A personalized diet based on DNA test results (2020-2022)<br>• Macronutrient/calorie counting (2020-2022)<br>• Mindful eating (2022)<br>• Intuitive eating (2022)<br>• Other [SPECIFY]<br>• No, have not followed any specific eating pattern or diet	Health; Environmental	2018-2022	"Vegetarian or vegan diet" was separated into two separate options: "vegetarian" and "vegan" for the 2020-2022 surveys.
Do you agree or disagree with the following statement? "It is hard for consumers to know whether the food choices they make are environmentally sustainable."	Scale from 1 (agree strongly) to 5 (disagree strongly)	Environmental	2019-2020	Response options 1 and 2 (i.e., agree strongly and agree) were combined for this analysis, as were 4 and 5 (i.e., disagree and disagree strongly)
Do you agree or disagree with the following statement? "If it was easier to know whether my food choices were environmentally sustainable, it	Scale from 1 (agree strongly) to 5 (disagree strongly)	Environmental	2019-2021	Response options 1 and 2 (i.e., agree strongly and agree) were combined for this analysis, as were

would have a greater influence on the choices I make.”				4 and 5 (i.e., disagree and disagree strongly)
You mentioned that it is important to you that food products are produced in an environmentally sustainable way. When shopping for foods and beverages, which of the following do you look for as a way to know that a product is produced in an environmentally sustainable way? Select all that apply.	• Recyclable packaging<br>• Minimal packaging (2019-2020)<br>• Reusable packaging (2021-2022)<br>• Labeled as organic<br>• Labeled as being locally grown<br>• Labeled as sustainably sourced<br>• Labeled as non-GMO/not bioengineered (BE)<br>• Labeled as bioengineered/containing bioengineered ingredients (asked 2020-2022)<br>• Other [SPECIFY]<br>• None of the above	Environmental	2019-2022	This question was only asked to individuals who responded "somewhat important" or "very important" to the question about whether it was important to know that the products they purchase or consume are produced in a sustainable way.
Do you generally try to consume or avoid the following?<br>• Animal protein<br>• Protein from plant sources	• Try to consume,<br>• Try to limit or avoid,<br>• Neither - just try to be aware,<br>• Don't consider,<br>• Not sure	Health; environmental	2019-2020	
Thinking about the last 12 months, how has your consumption of animal protein (e.g., meat, fish, poultry, dairy, eggs) and plant-based protein (e.g., soy/tofu, nuts, beans and legumes) changed?<br>• Animal-based protein (2019)<br>• Red meat (2020-2022)<br>• Poultry/eggs (2020-2022)<br>• Seafood (2020-2022)<br>• Dairy (2020–2022)<br>• Plant-based protein/protein from whole-plant sources (2019-2022 [slightly revised in 2022-see note])<br>• Plant-based meat alternatives/plant-based meat and seafood alternatives (2020- 2022)<br>• Plant-based dairy alternatives (2020-2022)<br>• Blended meat products (2022 only)<br>• Fortified soy-based milk and yogurt (2021-2022)	• Eat much more now<br>• Eat somewhat more now<br>• Eat the same amount<br>• Eat somewhat less now<br>• Eat much less now<br>• Never consume<br>• Not sure	Health; environmental	2020-2022	From 2020 onward, “animal-based protein” was changed to separate out red meat, poultry/eggs, and seafood.<br><br>“Plant-based protein” was revised to “protein from whole-plant sources” in 2022.<br><br>“Plant-based meat alternatives” was revised to “plant-based meat and seafood alternatives” in 2022.

				"Eat much more now" and "eat somewhat more now" were combined for this analysis, as were "eat somewhat less now" and "eat much less now"
To what degree do you believe your individual choices about food and beverage purchases impact the environment?	• My choices have a significant impact on the environment.<br>• My choices have a moderate impact on the environment.<br>• My choices have a small impact on the environment.<br>• My choices have no real impact on the environment.<br>• Not sure	Environmental	2021-2022	
Imagine you came across two food products that had the exact same Nutrition Facts panel. Would any of the following details lead you to believe that one of the products was more likely to be healthier?<br>• Product A is produced in a more environmentally sustainable way than Product B (2019-2021)<br>• Product A is described as a "plant-based food" on the label and Product B is not (2020-2022)<br>• Product A is described as "having a small carbon footprint" on the label and product B is not (2021-2022)	• Highly likely that Product A healthier<br>• Somewhat likely that Product A healthier<br>• Both products likely to be the same<br>• Somewhat likely that Product B healthier<br>• Highly likely that Product B healthier	Environmental	2019-2022	"Somewhat likely" and "highly likely" response options were combined for this analysis.
